# Sustainable Precursor-Based Titanium Dioxide–Graphene Nanocomposite Electrochemical Sensor for Sensitive Detection of Diuron in Vegetables

**DOI:** 10.3390/foods14172946

**Published:** 2025-08-24

**Authors:** Lisi Wang, Xiaoqing Li, Yijing Ai, Brij Mohan, Hongji Li, Zhisong Lu, Baoli Wang, Wei Sun

**Affiliations:** 1 Hainan International Joint Research Center of Marine Advanced Photoelectric Functional Materials, Key Laboratory of Laser Technology and Optoelectronic Functional Materials of Hainan Province, College of Chemistry and Chemical Engineering, Hainan Normal University, Haikou 571158, Chinahongjili1102@hainnu.edu.cn (H.L.); 2 Centro de Química Estrutural, Institute of Molecular Sciences, Instituto Superior Técnico, Universidade de Lisboa, Av. Rovisco Pais 1, 1049-001 Lisboa, Portugal; brij.mohan@tecnico.ulisboa.pt; 3 School of Materials and Energy/Institute for Clean Energy and Advanced Materials, Yibin Academy of Southwest University, Yibin 644000, China; 4 Haikou Key Laboratory of Marine Contaminants Monitoring Innovation and Application, Haikou Marine Geological Survey Center, Haikou 571158, China

**Keywords:** diuron, titanium dioxide, enteromorpha-derived carbon, laser-induced graphene electrode, food safety monitoring

## Abstract

The persistent presence of pesticide residues in vegetables raises significant concerns for food safety and public health, highlighting the need for sensing platforms that are efficient, affordable, and environmentally friendly while minimizing analysis time and reagent use. In this study, we developed a laser-induced graphene electrode (LIGE) modified with a titanium dioxide–Enteromorpha-derived carbon composite (TiO_2_@EDC) for the sensitive electrochemical detection of the herbicide diuron in vegetables. This integrated system streamlines material synthesis, electrode fabrication, and electrochemical analysis into a single, practical platform for food safety monitoring. Under optimized conditions, this sensor exhibited a wide linear detection range of 0.01 µM to 1 mM, with a low limit of detection of 2.99 nM (3 S/N) and a limit of quantification of 9.98 nM (10 S/N). Notably, the sensor demonstrated excellent analytical performance in real vegetable samples by accurately quantifying diuron residues in lettuce, indicating its potential for on-site monitoring of pesticide contamination in food matrices to ensure food safety.

## 1. Introduction

Diuron, a widely used herbicide, effectively controls weeds in agricultural and non-cultivated areas [1,2]. However, its chlorinated groups confer high toxicity, environmental persistence, and bioaccumulation potential, posing significant risks to ecosystems and food safety [3,4,5,6]. Recognized as a priority pollutant by the regulatory agencies of countries such as the U.S. and Canada, diuron demands reliable monitoring methods [7]. Conventional detection techniques—including high-performance liquid chromatography [8,9], gas chromatography [10], and mass spectrometry [11]—are often costly and lack portability. In contrast, electrochemical sensors offer a promising alternative due to their high sensitivity, selectivity, cost-effectiveness, portability, and potential for miniaturization in food safety applications [12,13,14].

Graphene (GR)-based electrochemical sensors have gained prominence owing to their exceptional physicochemical properties [15,16]. Among GR synthesis methods, laser-induced graphene (LIG) stands out as a solvent-free, high-efficiency approach that produces a three-dimensional porous structure with high conductivity and a large surface area [17,18,19,20]. These attributes make LIG ideal for sensing applications, as demonstrated in recent studies detecting bisphenol A, 4-nitrophenol, and chloramphenicol with high sensitivity [21,22,23].

To further enhance sensor performance, sustainable carbon sources, catalytic nanomaterials, and additive manufacturing techniques have been explored for modified electrode fabrication [24]. Enteromorpha, an abundant coastal algal waste, can be converted into porous activated carbon for applications in energy storage and electrochemical sensing [25]. When combined with titanium dioxide (TiO_2_), a material renowned for its stability, high electron mobility, and catalytic activity, the resulting nanocomposites significantly improve detection capabilities [26,27]. For instance, TiO_2_-activated carbon composites have been successfully employed in environmental pollutant monitoring. TiO_2_ can be used as an electrode modifier for electrochemical biosensors to detect various analytes. Pai et al. prepared activated carbon from betel nut shells and incorporated TiO_2_ to create a composite for modifying electrodes to detect diclofen in soil and water samples [28]. For example, Wu et al. used a nitrogen-doped Enteromorpha-derived porous carbon (EDC)-modified electrode for the catalytic analysis of p-nitrophenol [29].

In this study, we developed a flexible and integrated electrochemical sensor for the sensitive detection of diuron in food matrices. This sensor utilizes a laser-induced graphene electrode (LIGE) modified with TiO_2_@EDC. The LIGE is created through laser direct writing on polyimide, resulting in a conductive and defect-rich scaffold. The EDC, derived from pyrolyzed heteromorph, provides a mesoporous structure that enhances the adsorption of diuron. Additionally, the inclusion of TiO_2_ nanoparticles boosts the sensor’s catalytic activity. By combining this setup with a portable electrochemical workstation and a smartphone readout, we enable rapid, sensitive analysis of diuron residues in food samples. This offers a practical solution for food safety monitoring.

## 2. Experimental Section

### 2.1. Reagents

Diuron, glyphosate, and 3-indoleacetic acid were purchased from Shanghai Aladdin Reagent Co., Ltd. (Shanghai, China). PI film (0.1 mm) was obtained from Dongguan Kaipu Tape Co., Ltd. (Dongguan, China). Silver ink (01 L-2210) was acquired from Shenzhen Saiya Electronic Pulp Co., Ltd. (Shenzhen, China). Pluronic^®^ F127 (F127, PEO-PPO-PEO, tri-block copolymer) was obtained from Merck Chemical Technology Co., Ltd. (Shanghai, China). Titanium oxysulfate was obtained from Adamas Reagent Co., Ltd. (Shanghai, China). Nafion solution (5 wt. %) was obtained from Dupont Ltd. (Wilmington, DE, USA). Enteromorpha was collected from the coastal waters of Qingdao, China. All the reagents used were analytically pure, and all experiments were performed with ultrapure water filtered through a water purification system (Milli-Q IQ 7000, Merck Millipore, Darmstadt, Germany).

### 2.2. Instruments

Scanning electron microscopy (SEM) was performed using a Gemini SEM 300 microscope (ZEISS, Oberkochen, Germany), while transmission electron microscopy (TEM) images were acquired with a JSM-2100F microscope (JEOL, Tokyo, Japan). X-ray diffraction (XRD) patterns were recorded on an X’Pert3 diffractometer (PANalytical, Almelo, The Netherlands) using Cu Kα radiation (λ = 1.54 Å) over a 2θ range of 20–80°. X-ray photoelectron spectroscopy (XPS) analysis was conducted on an Escalab 250Xi spectrometer (Thermo Fisher, Waltham, MA, USA) with an Al Kα source (1486.6 eV), using the C1s peak (284.8 eV) as a reference for binding energy calibration. Raman spectra were obtained using a LabRAM HR Evolution spectrometer (Horiba, Villeneuve d’Ascq, France) with 514 nm excitation. Electrochemical measurements were carried out on an EmStat3 Blue workstation (Red Matrix Co., Ltd., Shenzhen, China), wirelessly controlled via a Huawei smartphone. Laser-induced graphene electrodes (LIGE) were fabricated using a Nano Pro III high-precision laser direct writing system (Tianjin Jiayin Nano Technology Co., Ltd., Tianjin, China).

### 2.3. Design and Fabrication of LIGE

**Scheme 1:**
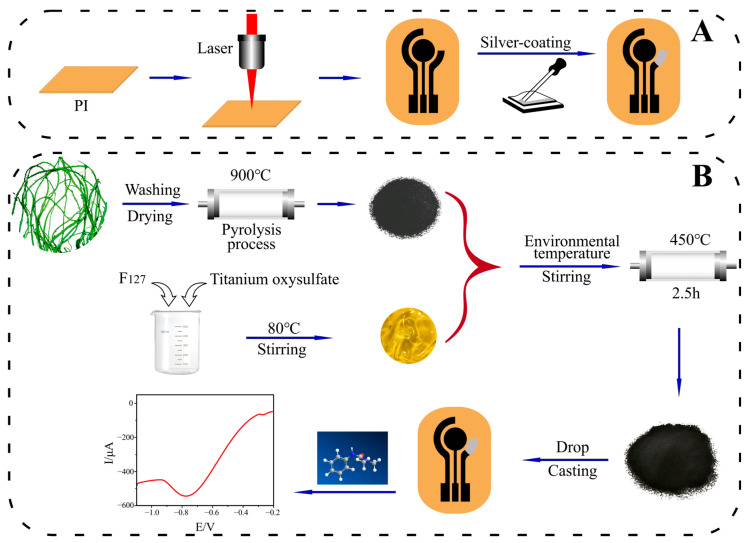
(**A**) The preparation process of LIGE; (**B**) the synthesis process of TiO_2_@EDC and electrochemical detection of diuron.

The PI film was rinsed sequentially with ethanol and ultrapure water, followed by air-drying. The electrode pattern was designed in FreeCAD (0.21.2) and then laser-etched onto the PI film using a Nano Pro-III laser direct writer (450 nm wavelength, 5.5 W max power, 10 μm direct writing accuracy) to form a graphene-based three-electrode (GR) structure. As illustrated in Scheme 1A, the LIGE was subsequently coated with conductive silver paste at one electrode end and oven-dried at 140 °C for 20 min prior to use.

### 2.4. Preparation of TiO_2_@EDC/LIGE

As depicted in Scheme 1B, fresh Enteromorpha was first washed with water and ethanol, then soaked in 0.01 mol/L hydrochloric acid solution for 24 h, followed by thorough rinsing with water and ethanol. After drying, it was carbonized at 900 °C for 2 h under an inert atmosphere to yield EDC (Enteromorpha-derived carbon). A sol was prepared by dissolving F127 as template and titanium sulfate as Ti source at 80 °C, followed by the addition of a controlled amount of EDC and continuous stirring at room temperature for 24 h. The obtained precursor was calcined at 450 °C for 2.5 h to produce TiO_2_@EDC. Finally, 6 μL of a well-dispersed TiO_2_@EDC suspension (2.0 mg/mL in water) was drop-cast onto the LIGE surface and air-dried, resulting in the TiO_2_@EDC/LIGE electrode.

### 2.5. Electrochemical Detection of Diuron

The electrochemical behavior of diuron was evaluated using cyclic voltammetry (CV) and chronocoulometry (CC) with a portable electrochemical workstation. CV and linear sweep voltammetry (LSV) measurements were performed in 0.1 M PBS (pH 7.0) over a potential window of −1.6 to 0.2 V. CC experiments employed the following parameters: potential range of −0.2 to 0.6 V with 0.25 s pulse width. Chronoamperometry (CA) was conducted at −0.9 V initial potential with 0.1 s sampling interval for 30 s duration. All electrochemical measurements were performed at ambient temperature and pressure.

### 2.6. Real Sample Analysis

Fresh lettuce samples (5.0 g), obtained from a local market, were homogenized with 5 mL of methanol for 10 min. The extract was centrifuged at 4000 rpm for 10 min, and the resulting supernatant was filtered through a 0.22 µm membrane filter. The filtrate was then diluted 100-fold with PBS (pH 7.0) prior to analysis. The prepared samples were analyzed for diuron content under optimized experimental conditions using the developed method.

## 3. Results and Discussion

### 3.1. Characterization

**Figure 1:**
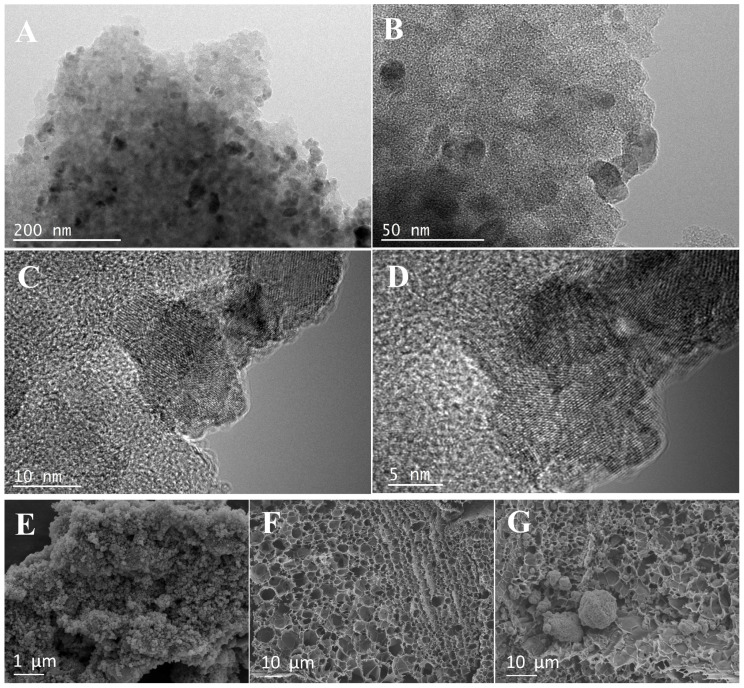
TEM (**A**,**B**) and HRTEM (**C**,**D**) of TiO_2_@EDC with different magnifications; SEM of (**E**) TiO_2_@EDC, (**F**) LIGE, and (**G**) TiO_2_@EDC/LIGE.

TEM and high-resolution TEM (HRTEM) analyses revealed the morphological and crystalline characteristics of the composite material. As shown in Figure 1A,B, TiO_2_ nanoparticles with uniform particle sizes of 10–20 nm were well-dispersed on the EDC substrate without apparent agglomeration. HRTEM images (Figure 1C,D) clearly displayed the characteristic lattice fringes of both the curved graphitic structure of EDC and the crystalline TiO_2_ phases, with visible interplanar spacings corresponding to anatase and rutile TiO_2_, confirming successful composite formation. Complementary SEM characterization (Figure 1E) further verified the homogeneous distribution of TiO_2_ nanoparticles on the EDC surface. Figure 1F demonstrates the successful laser-induced conversion of PI film into GR, as evidenced by the distinct honeycomb lattice structure characteristic of GR materials [30]. The three-dimensional porous architecture of laser-induced graphene GR provided LIGE with an enhanced specific surface area while promoting rapid electron transfer kinetics. As evidenced in Figure 1G, TiO_2_@EDC composites (~10 μm in diameter) were uniformly distributed across the conductive GR framework. This hierarchical structure, combining the high surface area of GR with the electrocatalytic properties of TiO_2_@EDC, created an optimal platform for electrochemical sensing applications.

The XRD pattern of the TiO_2_@EDC composite reveals several distinct peaks (Figure S1). The peaks at 25.3°, 36.9°, 37.8°, 48.0°, 53.9°, 55.0°, 62.7°, 68.8°, 70.3°, and 75.0° correspond to the diffraction peaks of anatase TiO_2_, associated with the crystal planes (101), (103), (004), (200), (105), (211), (204), (116), (220), and (215). Additionally, the peaks at 27.4° and 36.1° are attributed to the (110) and (101) crystal faces of rutile TiO_2_ [31], and the peaks at 26.5° and 42.1° correspond to the (002) and (110) crystal faces of graphite carbon, indicating that the TiO_2_ in the TiO_2_@EDC composite is a mixture of anatase and rutile phases.

The Raman spectrum of TiO_2_@EDC (Figure S2) exhibited characteristic peaks at 1322 cm^−1^ (D band) and 1580 cm^−1^ (G band), corresponding to disordered carbon and graphitized carbon, respectively. The I_D_/I_G_ intensity ratio of ~1.3 suggests defect formation in the EDC, which could enhance active site availability. Additionally, peaks at 422 cm^−1^ and 608 cm^−1^ were attributed to rutile-phase TiO_2_, while those at 139 cm^−1^ and 255 cm^−1^ were assigned to anatase-phase TiO_2_. These findings confirm the coexistence of both rutile and anatase phases in the composite, aligning well with the XRD results [32].

X-ray photoelectron spectroscopy (XPS) analysis was conducted to investigate the elemental composition of TiO_2_@EDC. In the Ti 2p spectrum (Figure S3A), the peaks observed at 458.9 eV (Ti 2p_3_/_2_), 464.6 eV (Ti 2p_1_/_2_), and 472.2 eV (satellite peak) confirm the presence of Ti^4+^ in TiO_2_. The O 1s spectrum (Figure S3B) displays three contributions: 530.2 eV (lattice oxygen in TiO_2_), 531.0 eV (oxygen defects), and 532.4 eV (adsorbed oxygen species, such as O_2_^−^ and OH^−^). These findings indicate the presence of surface oxygen functionalities.

To further investigate the pore structure of TiO_2_@EDC, N_2_ adsorption–desorption measurements were performed. As shown in Figure S4A, the sample exhibited a Type IV isotherm with an H3-type hysteresis loop, confirming its mesoporous nature. The specific surface area was calculated to be 99.595 m^2^/g, while the pore size distribution curve (Figure S4B) revealed an average pore diameter of 3.818 nm, likely originating from the carbon matrix. This hierarchical porous structure facilitates efficient adsorption and diffusion of electrons and ions, enhancing electrochemical performance.

### 3.2. Electrochemical Behaviors of Different Electrodes

**Figure 2:**
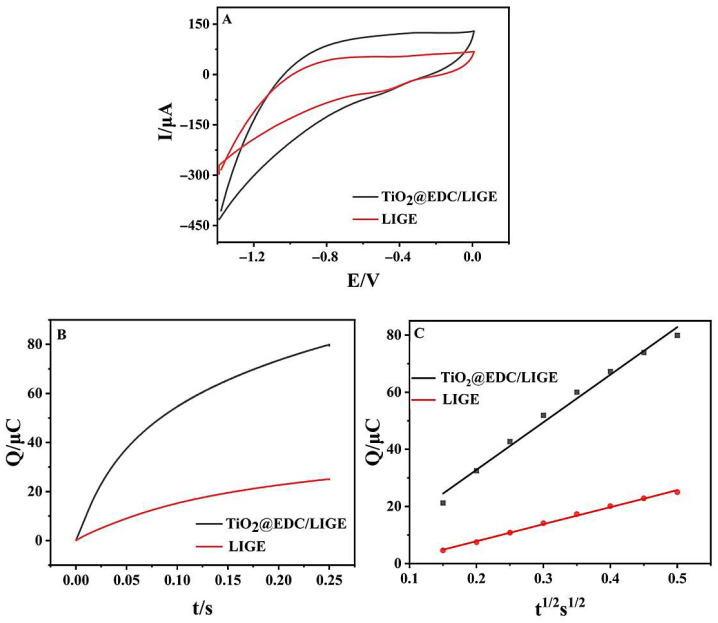
(**A**) Electrochemical behaviors of different electrodes in 0.1 mol/L PBS (pH 7.0); (**B**) Q-t curves of different electrodes in a solution of 1.0 mM K_3_[Fe(CN)_6_] and 0.5 M KCl; and (**C**) the linear relationship between Q and t^1/2^ of different electrodes.

As illustrated in Figure 2A, the CV curves of TiO_2_@EDC/LIGE and LIGE in pH 7.0 PBS were recorded without any observable redox peaks, indicating that no redox reactions occurred on the electrode surface. CC was carried out in a mixed solution of 1.0 mmol/L K_3_[Fe(CN)_6_] and 0.5 mol/L KCl to assess the effective electrode area of different working electrodes. The relationship between the absolute charge variations against t and t^1/2^ for TiO_2_@EDC/LIGE and LIGE was shown in Figure 2B,C. There was a linear relationship between the charge and the square root of time, with the linear regression equation for TiO_2_@EDC/LIGE as Q (μC) = 166.553 t^1/2^ (s^1/2^) − 0.472 (R^2^ = 0.9885) and for LIGE as Q (μC) = 59.655 t^1/2^ (s^1/2^) − 4.113 (R^2^ = 0.9962). To obtain the effective surface area, the Anson equation [33] was utilized as follows: Q = $\frac{2nFAc{\left(Dt\right)}^{1/2}}{\pi^{1/2}}$ + Q_dl_ + Q_ads_. The effective surface areas of TiO_2_@EDC/LIGE and bare LIGE were 0.550 cm^2^ and 0.197 cm^2^. The increase in the effective area of the TiO_2_@EDC/LIGE electrode can be attributed to the honeycomb-like structure of the TiO_2_@EDC nanocomposite, which possesses a large specific surface area with high roughness.

### 3.3. Electrochemical Performance and Kinetics for Diuron Detection

**Figure 3:**
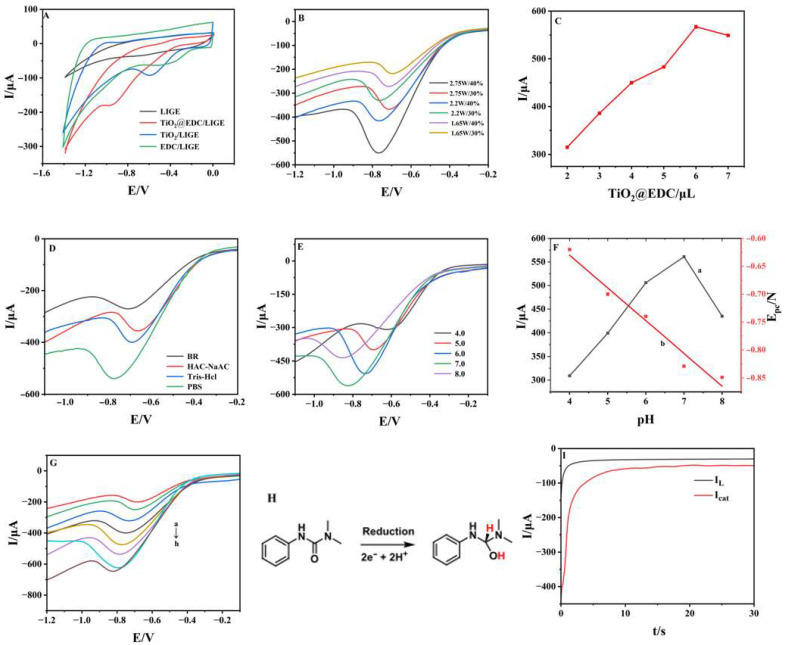
(**A**) Electrochemical behaviors of 0.02 mmol/L diuron at different electrodes in 0.1 mol/L PBS (pH 7.0); effects of (**B**) different laser powers and engraving depth; (**C**) different volume of 2.0 mg/mL TiO_2_@EDC on electrode output signal; LSV of 1.0 mmol/L diuron at TiO_2_@EDC/LIGE in (**D**) 0.1 mol/L BR, HAC-NaAC, Tris-HCl, PBS, and (**E**) different pH values of PBS (a→e: pH 4.0→8.0); (**F**) the plot of Ipc (a) and Epc (b) of diuron versus pH; (**G**) LSV of TiO_2_@EDC/LIGE at different scan rates (a-i: 0.01, 0.02, 0.04, 0.06, 0.08, 0.1, 0.20, 0.25 V/s) in 1.0 mmol/L diuron and 7.0 PBS; (**H**) electrochemical reduction mechanism of diuron; (**I**) CA response of TiO_2_@EDC/LIGE in 0.1 mol/L PBS (pH 7.0) in the absence (I_L_) and presence (I_cat_) of 1.0 mmol/L diuron.

The electrochemical behavior of 0.02 mmol/L diuron was investigated in 0.1 mmol/L PBS (pH 7.0) using cyclic voltammetry (CV) to investigate electrocatalytic activity and optimization. As shown in Figure 3A, the TiO_2_@EDC/LIGE exhibited a significantly higher reduction peak current than bare LIGE, confirming the composite’s ability to enhance the electroreduction of diuron. To further elucidate the individual contributions of TiO_2_ and EDC, control experiments were conducted using TiO_2_/LIGE and EDC/LIGE for comparison. The reduction peak currents followed the order of TiO_2_@EDC/LIGE > TiO_2_/LIGE > EDC/LIGE > bare LIGE. This trend demonstrates that both TiO_2_ and EDC individually improve the electrochemical performance. Among them TiO_2_ offers high catalytic activity, chemical stability, and rapid electron transfer, while EDC provides a mesoporous carbon framework with a large surface area, facilitating diuron adsorption and diffusion. When combined, TiO_2_ nanoparticles are uniformly distributed across the EDC’s porous network, leading to (i) an increased density of electroactive sites, (ii) shortened electron transport pathways, and (iii) enhanced analyte enrichment at the electrode surface. This improvement is attributed to the TiO_2_@EDC modification, which provides a more electrochemically active interface with increased catalytic sites. The effects of laser power and engraving depth on electrode performance were systematically studied (Figure 3B). The optimal conditions were found at 2.75 W laser power and 40% engraving depth, yielding the highest peak current. Deviations from these parameters, such as either insufficient laser energy leading to incomplete graphene formation or excessive power/depth causing mechanical damage, resulted in diminished electrode performance.

To investigate the effect of TiO_2_@EDC loading, the influence of TiO_2_@EDC drop-coating volume (2–7 µL) on the reduction peak current is presented in Figure 3C. The current increased with loading up to 6 µL, beyond which it declined due to excessive composite thickness, hindering mass transport. Thus, 6 µL was selected as the optimal loading. Four buffer systems (HAc-NaAc, Tris-HCl, BR, and PBS) were evaluated for diuron detection (Figure 3D). PBS (0.1 M) demonstrated the highest sensitivity in linear sweep voltammetry (LSV) and was chosen as the optimal medium. Additionally, pH dependence and reaction mechanism were investigated as the electrochemical reduction of diuron was highly pH-dependent (Figure 3E). The reduction peak shifted negatively from –0.620 V (pH 4.0) to –0.849 V (pH 8.0), indicating proton involvement in the redox process. A linear relationship between peak potential (Epc) and pH was established (Figure 3F): Epc (V) = –0.058 pH − 0.395 (R^2^ = 0.9852). The slope at 58 mV/pH closely matches the theoretical Nernstian value (59 mV/pH), suggesting an equal number of protons and electrons in the diuron reduction mechanism. The peak current reached its maximum at pH 7.0, beyond which it decreased, likely due to hydroxyl-induced ring-opening degradation of diuron at alkaline conditions (pH > 7.0). Therefore, pH 7.0 PBS was selected for subsequent experiments [34].

The electrochemical behavior of 1.0 mmol/L diuron at TiO_2_@EDC/LIGE was investigated across scan rates from 10 to 250 mV/s (Figure 3G). The reduction peak current exhibited a linear dependence on the square root of scan rate (υ^1^/^2^): Ipc (μA) = −1169.973 υ^1^/^2^ (V/s) − 103.329 (R^2^ = 0.9808). This relationship confirms a diffusion-controlled process [35], where mass transport dominates the electrochemical reduction. Concurrently, the peak potential shifted negatively with increasing scan rate, following the linear relationship Epc (V) = 0.0326 lnυ − 0.853 (R^2^ = 0.9851). This behavior indicates an irreversible electron transfer process. Using Laviron’s equation [36], Ep = (RT/αnF)lnυ, where R is the gas constant (8.314 J·mol^−1^·K^−1^); α, the transfer coefficient (0.5); F, Faraday’s constant (96,485 C·mol^−1^); and T, the temperature (298 K), we determined *n* ≈ 2. This two-electron reduction corresponds to carbonyl group conversion to a hydroxyl group [36], with the proposed mechanism illustrated in Figure 3H [37]. To investigate catalytic activity analysis, chronoamperometric studies at −0.9 V revealed enhanced current (Icat) in the presence of 1.0 mmol/L diuron compared to the background (IL) (Figure 3I). The linear Icat/IL vs. t^1^/^2^ relationship, Icat/IL = 3.725 t^1^/^2^ (s^1^/^2^) + 2.584 (R^2^ = 0.9760), yielded a catalytic rate constant (kcat) of 4.419 × 10^3^ M^−1^·s^−1^ through the equation Icat/IL = (πkcatC_0_t)^1^/^2^ [38]; this substantial kcat value demonstrates the exceptional catalytic activity of the TiO_2_@EDC/LIGE interface for diuron reduction.

### 3.4. Analytical Performances

**Figure 4:**
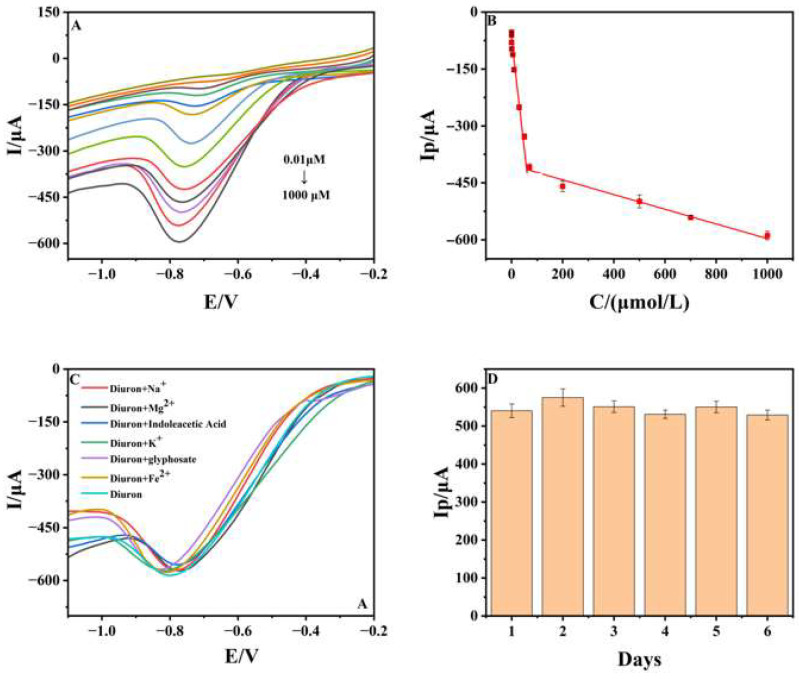
(**A**) LSV of TiO_2_@EDC/LIGE in different concentrations of diuron (0.01, 0.05, 0.10, 1.00, 5.00, 10.00, 30.00, 50.00, 70.00, 200.00, 500.00, 700.00, 1000.00 μmol/L); (**B**) the relationship of the reduction peak currents with diuron concentrations; (**C**) the peak currents of TiO_2_@EDC/LIGE for the detection of 1.0 mmol/L diuron with the addition of the different interfering agents in pH 7.0 PBS; (**D**) the reduction current values of TiO_2_@EDC/LIGE in 1.0 mmol/L diuron for 6 days.

Under optimized conditions, the LSV response of diuron on the TiO_2_@EDC/LIGE sensor was recorded (n = 3), as shown in Figure 4A. The peak current increased proportionally with rising diuron concentrations. A linear calibration curve (Figure 4B) was obtained over two concentration ranges, 0.01–70.0 μM and 70.0–1000.0 μM, with corresponding regression equations of Ip (μA) = −4.901C (μmol/L) − 80.960 (R^2^ = 0.9801) and Ip (μA) = −0.184C (μmol/L) − 408.521 (R^2^ = 0.9812). The sensor exhibited a wide linear detection range from 10 µM to 1 mM, with a low limit of detection (LOD) of 2.99 nM (3 S/N) and a limit of quantification (LOQ) of 9.98 nM (10 S/N). As summarized in Table 1, the analytical performance of the proposed sensor was compared with previously reported methods, demonstrating superior sensitivity and a broad detection range. The sensor’s anti-interference capability was evaluated using common interferents, including 10.0 mmol/L Na^+^, Mg^2+^, K^+^, and Fe^2+^, and 2.0 mmol/L of 3-indoleacetic acid and glyphosate. As illustrated in Figure 4C, these substances had negligible effects on the reduction current of diuron, confirming the excellent anti-interference capability of the TiO_2_@EDC/LIGE sensor. Stability tests were performed by storing the sensor at room temperature and measuring the LSV peak current in a 1.0 mmol/L diuron solution over six days (n = 3). As shown in Figure 4D, minimal current variation was observed with relative standard deviation (RSD) values below 5.00%, indicating the sensor exhibits excellent long-term stability. Additionally, intra-day reproducibility was evaluated by testing five independently fabricated electrodes (Figure S5A), which showed RSD values below 5.00% for all electrodes (1.4%, 0.93%, 2.48%, 0.95%, and 0.64%), demonstrating good fabrication consistency and reliability.

### 3.5. Real Sample Detection

The practical applicability of the electrochemical sensor was further evaluated using lettuce as a real sample. Diuron content was determined using LSV based on the proposed procedure and the standard addition method. For each spiking level, three independent measurements were performed (n = 3); RSD and recovery values were calculated to evaluate precision and accuracy. The results, presented in Table 2, showed recovery rates ranging from 98.14% to 101.69% with low RSD values (<5.00%), demonstrating the sensor’s reliability and suitability for detecting diuron in real-world samples.

## 4. Conclusions

This study successfully developed a high-performance electrochemical sensor based on a LIGE modified with a titanium dioxide/Enteromorpha-derived carbon (TiO_2_@EDC) composite for the sensitive and selective detection of diuron residues in food matrices. The synergistic integration of the 3D porous graphene scaffold and the TiO_2_@EDC nanocomposite significantly enhanced the electrode’s electrochemical activity, providing a large surface area, improved electron transfer, and abundant catalytic sites. The sensor exhibited outstanding analytical performance, including a wide linear detection range of 0.01–1000.0 μmol/L, LOD of 2.99 nM, LOQ of 9.98 nM, excellent anti-interference capability, and remarkable stability. Real-sample analysis in lettuce validated its practical applicability, yielding 98.14% and 101.69% recovery rates. This work presents a cost-effective, environmentally friendly, and portable sensing platform with promising potential for on-site, real-time monitoring of diuron and other pesticide residues in complex food matrices, contributing to food safety assurance.

## Figures and Tables

**Table 1:** Comparison of different electrochemical detection methods for diuron.

Modified Electrodes	Method	Sample	Linear Range (μmol/L)	LOD (μmol/L)	Ref.
rGO/AuNPs/ SPE	LSV	Lake water and sea water	2.15–128.7	0.54	[35]
MWCNT- COOH/GCE	DPV	Sea water	0.215–2.15	0.068	[39]
MWCNT- COOH-MIP/ CPE	SWV	River water	0.05–1.25	0.009	[40]
p-NiTSPc/ CFME	SWV	A commercialized formulation of diuron	21.45–150	8.03	[41]
NiOPc/hemin/GO/CPE	FIA/Amperometry	Soil and carrot	5.00–140	1.72	[42]
BaO-MWCNT/ MCPE	AMP i-t curve	Soil and water	1–100	0.310	[43]
ZnONPs/CPE	CV	Soil and water	1.3–7.7, 8.6–30	0.223	[44]
TiO_2_@EDC/ LIGE	LSV	Lettuce	0.01–1000	2.99 × 10^−3^	This work

Note: MWCNT: rGO–AuNP: reduced graphene oxide–gold nanoparticle; SPE: screen-printed electrode; multi-walled carbon nanotube; GCE: glassy carbon electrode; MWCNT-COOH-MIP/CPE: carbon paste electrodemodified with a molecularly imprinted polymer and carboxyl functionalized multiwalled carbon nanotubes; rGO–AuNP p-NiTSPc: Nickel(II) tetrasulfonated phthalocyanine; CFME: carbon fiber microelectrode; CPE/NiOPc/hemine/GO: Carbon paste electrode/nickel (l) 1,48, 11, 15, 18, 22, 25-octabutoxy-29H, 31H-phthalocyanine complex; BaO-MWCNT: carbon nanotube decorated barium-oxide; MCPE: modified carbon paste electrode. ZnONPs-CPE: Zinc oxide nanoparticles/modified carbon paste electrode.

**Table 2:** Diuron estimation in lettuce sample using TiO_2_@EDC/LIGE (n = 3).

Sample	Added (µM)	Found (µM)	RSD (%)	Recovery (%)
Lettuce	0	0	-	-
	6.50	6.61	4.96	101.69
	10.50	10.53	3.52	100.29
	14.50	14.23	1.11	98.14

## Data Availability

The original contributions presented in the study are included in the article and Supplementary Material, further inquiries can be directed to the corresponding authors

## Supplementary Materials

The following supporting information can be downloaded at https://www.mdpi.com/article/10.3390/foods14172946/s1: Figure S1: XRD pattern TiO_2_@EDC; Figure S2: Raman spectrum of TiO_2_@EDC; Figure S3: XPS spectra of (**A**) Ti 2p and (**B**) O 1s of TiO2@EDC; Figure S4: (**A**,**B**) Nitrogen isothermal adsorption–desorption curves and pore size distribution curves of TiO_2_@EDC; Figure S5: (A) Reproducibility studies of the sensor.
