# Supplementary material for: Sustainable Precursor-Based Titanium Dioxide–Graphene Nanocomposite Electrochemical Sensor for Sensitive Detection of Diuron in Vegetables
Source: Foods. 2025 Aug 24;14(17):2946. doi: 10.3390/foods14172946 (PMC12428406; doi:10.3390/foods14172946)
Supplement: Supplementary file 1 [file foods-14-02946-s001.zip › foods-3806709-supplementary.pdf]

Sustainable Precursor-Based Titanium Dioxide–Graphene Nanocomposite Electrochemical Sensor for Sensitive Detection of Diuron in Vegetables

Lisi Wang[a], Xiaoqing Li[a], Yijing Ai[a], Brij Mohan[b], Hongji Li[a], Zhisong Lu[c], Baoli Wang[d,*] and Wei Sun[a,*]

[a]Hainan International Joint Research Center of Marine Advanced Photoelectric Functional Materials, Key Laboratory of Laser Technology and Optoelectronic Functional Materials of Hainan Province, College of Chemistry and Chemical Engineering, Hainan Normal University, Haikou 571158, P. R. China

[b]Centro de Química Estrutural, Institute of Molecular Sciences, Instituto Superior Técnico, Universidade de Lisboa, Av. Rovisco Pais 1, 1049-001 Lisboa, Portugal

[c] Yibin Academy of Southwest University, Yibin 644000, P. R. China

[d]Haikou Key Laboratory of Marine Contaminants Monitoring Innovation and Application, Haikou Marine Geological Survey Center, Haikou 571158, P. R. China

Emails: sunwei@hainnu.edu.cn (W. Sun), baoli_0916@163.com (B. L. Wang)

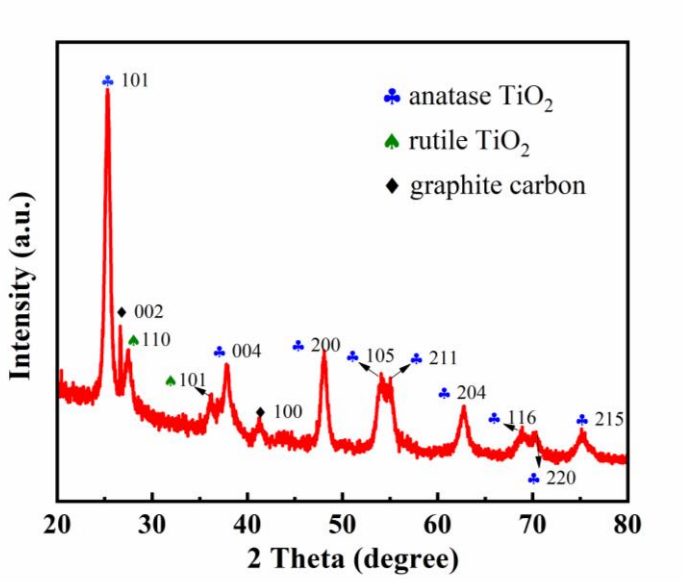

Figure S1. XRD pattern TiO_2@EDC.

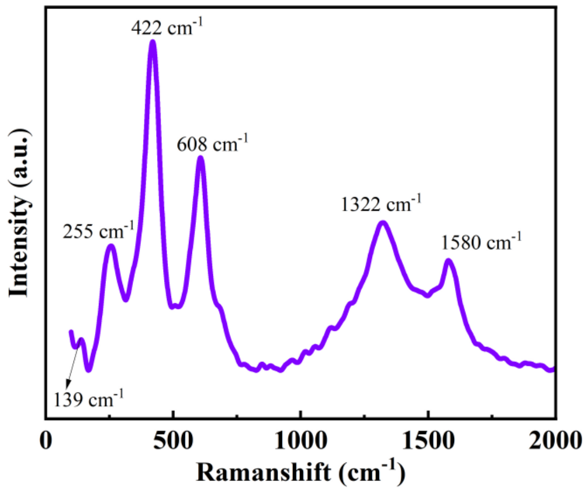

Figure S2. Raman spectrum of TiO_2@EDC.

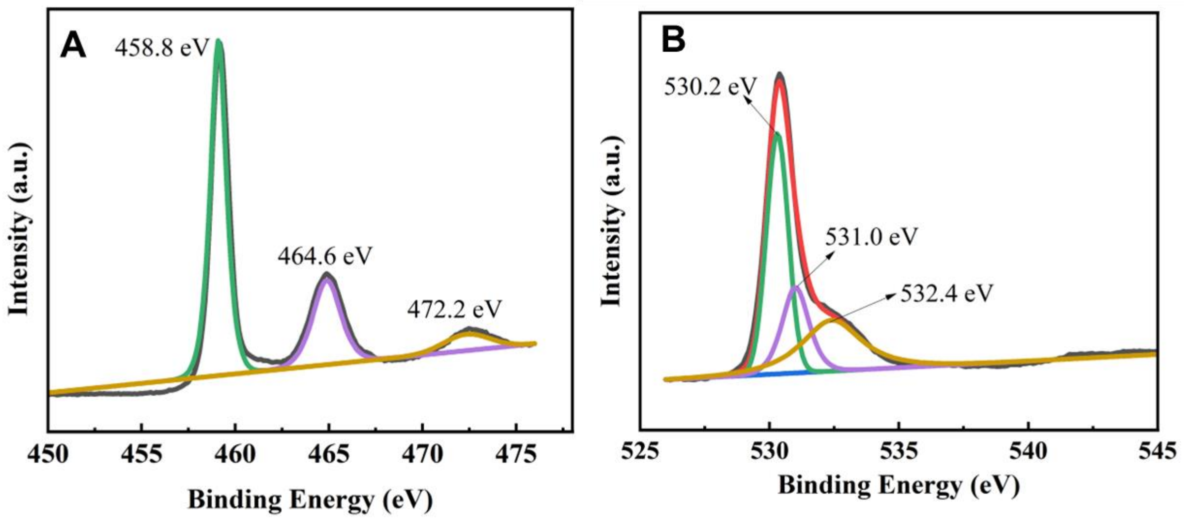

Figure S3. XPS spectra of (A) Ti 2p and (B) O 1s of TiO_2@EDC.

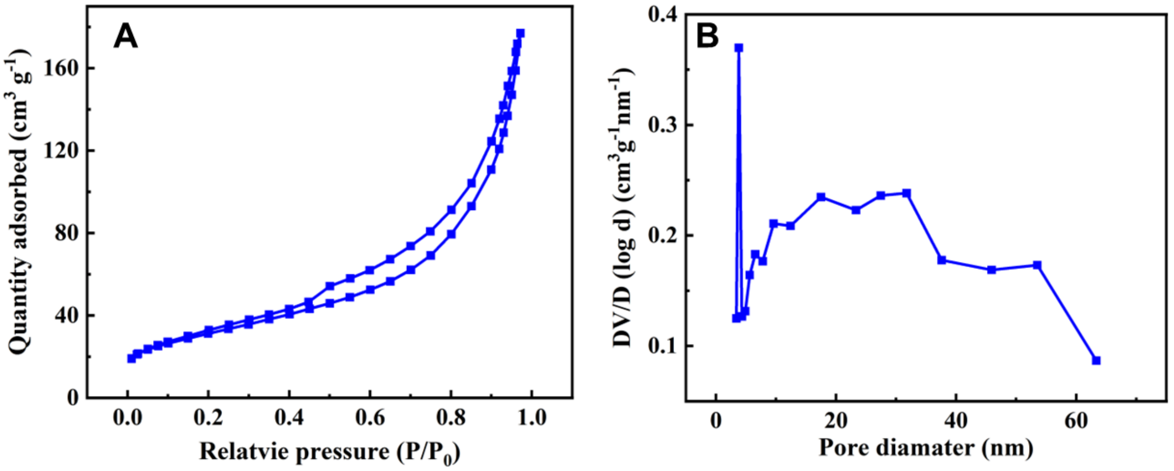

Figure S4. (A, B) Nitrogen isothermal adsorption–desorption curves and pore size distribution curves of TiO_2@EDC.

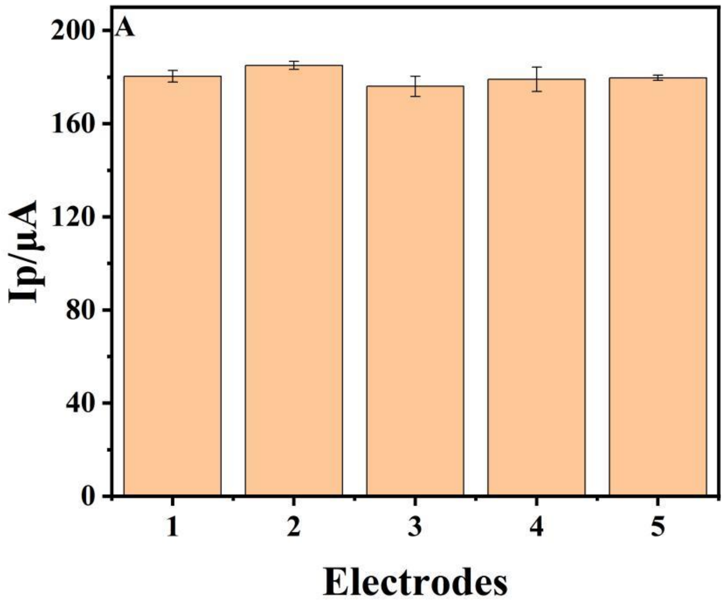

Figure S5. (A) Reproducibility studies of the sensor using 0.02 mmol/L diuron in 0.1 mol/L PBS (pH 7.0).
